# Supplementary material for: Enhanced Notch dependent gliogenesis and delayed physiological maturation underlie neurodevelopmental defects in Lowe syndrome
Source: EMBO Mol Med. 2025 Nov 11;17(12):3407–39. doi: 10.1038/s44321-025-00327-y (PMC12686420; doi:10.1038/s44321-025-00327-y)
Supplement: Supplementary file 2 — Movie EV1 [file 44321_2025_327_MOESM2_ESM.zip › MovieEV1/readme.rtf]

Timelapse calcium imaging of WT1 neurons showing calcium transients, scale bar 50um. Basal calcium activity is recorded till 4mins, followed by addition of TTX and neurons are depolarised with high KCL at 8th min. Movie is sped up to 40fps.
