# Supplementary material for: Enhanced Notch dependent gliogenesis and delayed physiological maturation underlie neurodevelopmental defects in Lowe syndrome
Source: EMBO Mol Med. 2025 Nov 11;17(12):3407–39. doi: 10.1038/s44321-025-00327-y (PMC12686420; doi:10.1038/s44321-025-00327-y)
Supplement: Supplementary file 6 — Expanded View Figures [file 44321_2025_327_MOESM6_ESM.pdf]

Expanded View Figures

Figure EV1. Generation of OCRL^{KO} cell line.

(**A**) iPSC-derived NSCs using dual-SMAD inhibition exhibit canonical NSC markers Nestin (red), Pax6 and FOXG1 (green). Nuclei were stained with DAPI, Scale bar = 50 µm. Immunofluorescence images obtained from WT1, LSP2, LSP3 and LSP4. (**B**) CRISPR-Cas9 targeting strategy: Two sgRNAs (G1 and G2) were designed to target exon 8 of *OCRL*, prior to the 5′-Phosphatase domain of the protein. Target sites of *OCRL*-688-G1 and *OCRL*-688-G2 are highlighted in yellow and green, respectively. (**C**) Sequence chromatogram confirming insertion of 11 bp (red outlined box) in OCRL^{KO} iPSC (lower panel), compared to non-edited WT2 iPSC (upper panel). (**D**) iPSC from WT2 and OCRL^{KO} displaying pluripotent markers viz., SOX2 (green), SSEA4 (red), Oct-4 (green), TRA-160 (red); iPSCs were counter-stained with DAPI (blue), scale bar = 50 µm. (**E**) Normal karyogram confirming chromosomal integrity of OCRL^{KO} iPSC. (**F**) iPSC-derived NSC from WT2 and OCRL^{KO} exhibit canonical NSC markers Nestin (red), Pax6 and FOXG1 (green). Nuclei were stained with DAPI, scale bar = 50 µm.

 © The Author(s)

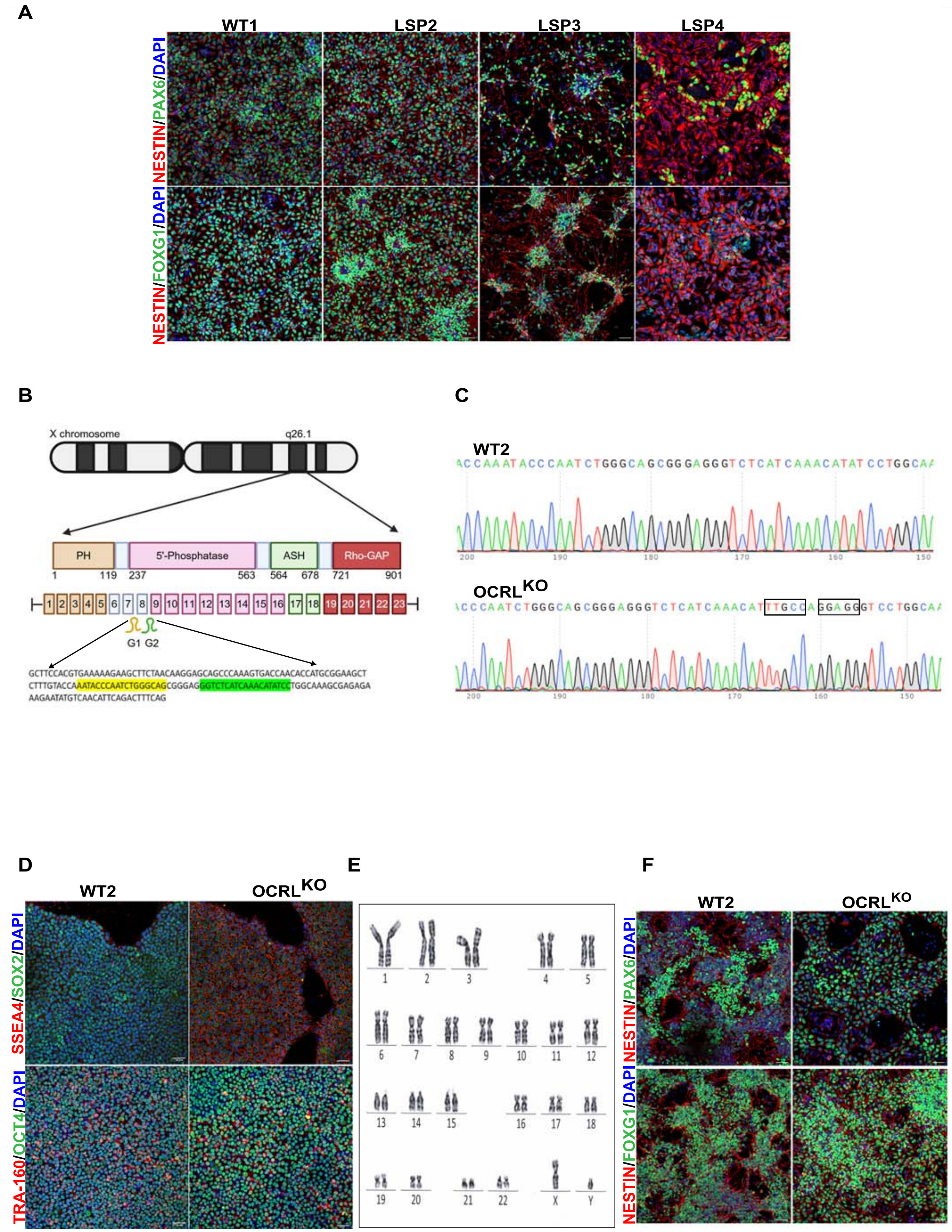
A
WT1
LSP2
LSP3
LSP4
NESTIN/PAX6/DAPI
NESTIN/FOXG1/DAPI
B
X chromosome
q26.1
PH
5'-Phosphatase
ASH
Rho-GAP
1
119
237
563
564
678
721
901
1 2 3 4 5 6 7 8 9 10 11 12 13 14 15 16 17 18 19 20 21 22 23
G1 G2
GCTTCCACGTGAAAAAGAAGCTTCTAACAAGGAGCAGCCCAAAGTGACCAACACCATGCGGAAGCT
CTTTGTACCAAATACCCAATCTGGGCAGCGGGAGGGTCTCATCAAACATATCCTGGCAAAGCGAGAGA
AAGAATATGTCAACATTCAGACTTTCAG
C
WT2
CCAAATACCCAATCTGGGCAGCGGGAGGGTCTCATCAAACATATCCTGGCA
200
190
180
170
160
150
OCRL^KO
CCCAATCTGGGCAGCGGGAGGGTCTCATCAAACATTTGCCAGGAGGGTCCTGGCA
200
190
180
170
160
150
D
WT2
OCRL^KO
SSEA4/SOX2/DAPI
TRA-160/OCT4/DAPI
E
1 2 3 4 5
6 7 8 9 10 11 12
13 14 15 16 17 18
19 20 21 22 X Y
F
WT2
OCRL^KO
NESTIN/PAX6/DAPI
NESTIN/FOXG1/DAPI

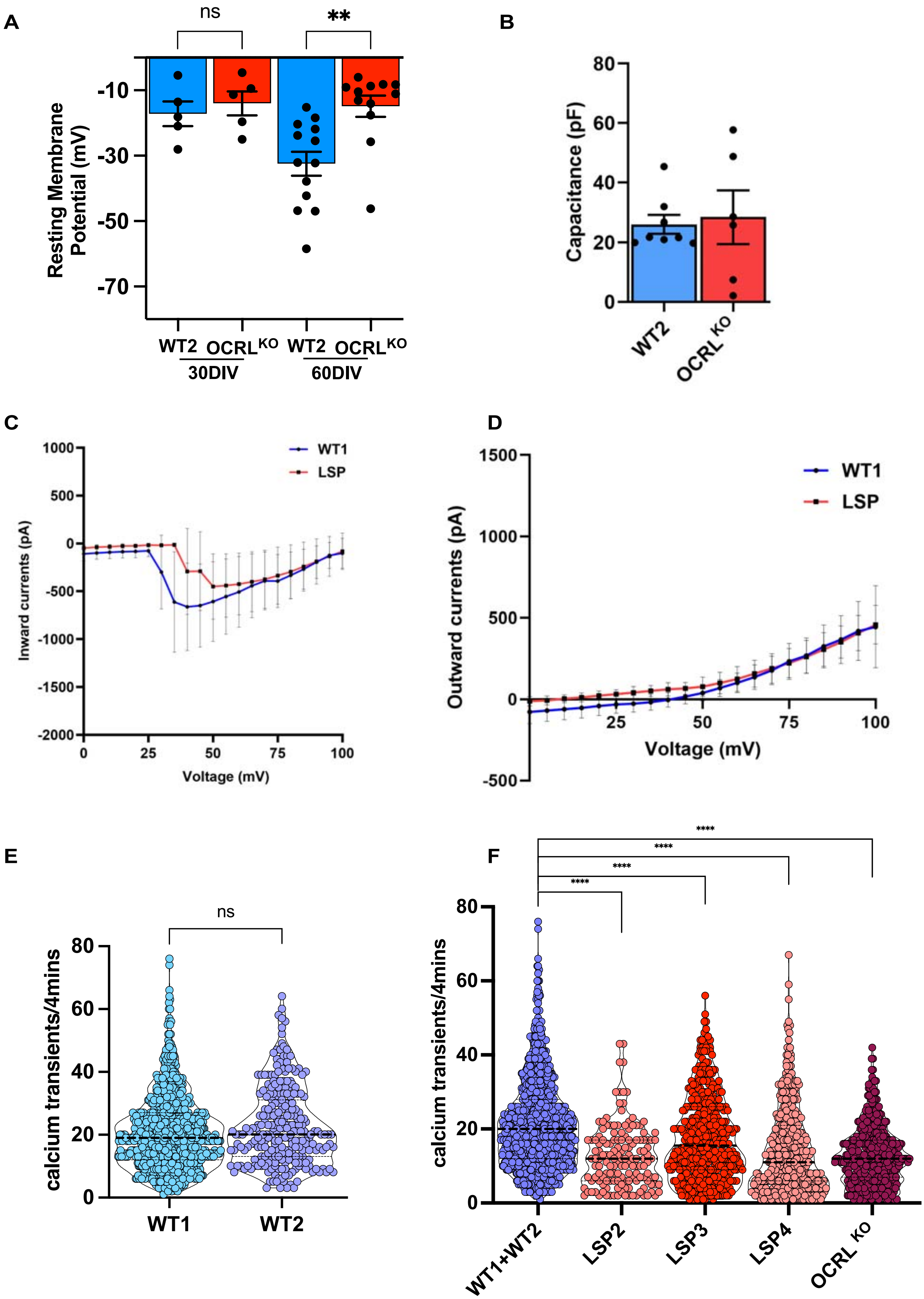
A
ns
**
Resting Membrane Potential (mV)
-10
-30
-50
-70
WT2 OCRL KO WT2 OCRL KO
30DIV 60DIV
B
Capacitance (pF)
80
60
40
20
0
WT2
OCRL KO
C
WT1
LSP
Inward currents (pA)
1000
500
0
-500
-1000
-1500
-2000
0 25 50 75 100
Voltage (mV)
D
WT1
LSP
Outward currents (pA)
1500
1000
500
0
-500
25 50 75 100
Voltage (mV)
E
ns
calcium transients/4mins
80
60
40
20
0
WT1
WT2
F
****
****
****
****
calcium transients/4mins
80
60
40
20
0
WT1+WT2
LSP2
LSP3
LSP4
OCRL KO

Figure EV2. Physiological properties of iPSC derived neurons.

(**A**) Resting membrane potential from WT2 and $OCRL^{KO}$ derived from 30 and 60 DIV neurons measured during whole cell recordings. Y-axis show resting membrane potential in mV. Each point represents recording from a single neuron obtained through multiple differentiations ($n = 2$). Statistical significance was assessed using ordinary one-way ANOVA followed by Tukey's multiple comparison test, $OCRL^{KO}$ vs WT2 60DIV $p = 0.0043$**. Error bars: Mean +/− SEM shown. (**B**) Capacitance measurements from WT2 and $OCRL^{KO}$ 60 DIV neurons measured during whole cell recordings ($n = 2$). Y-axis shows capacitance in pF. Each point represents a single cell. Error bars: Mean +/− SEM. Whole-cell patch clamp recordings in voltage-clamp mode from 60DIV WT1 and pooled LSP neurons showing ($n = 2$): (**C**) Inward and (**D**) outward currents. Y-axis shows currents in pA. X-axis voltage in mV. Averaged traces from WT1 (11 cells) and LSP (6 cells) is shown ($n = 2$). Current amplitude at each voltage is shown as mean +/− SEM. (**E**) $[Ca^{2+}]_i$ transients data comparing two control cell lines WT1 and WT2 show no statistical difference in the frequency of calcium transients (WT1 $n = 5$; WT2 $n = 2$). Statistical significance was assessed by using Mann–Whitney t-test. (**F**) WT1 and WT2 combined using the available data and plotted against three individual LS patient lines and $OCRL^{KO}$ (WT1, LSP2, LSP3, $n = 3$; WT1, LSP4, WT2, $OCRL^{KO}$ $n = 2$). Statistical test used: one-way ANOVA followed by Dunnett's multiple comparison test, WT1 + WT2 vs LSP2, WT1 + WT2 vs LSP3, WT1 + WT2 vs LSP4, WT1 + WT2 vs $OCRL^{KO}$ $p = <0.0001$****.

© The Author(s)

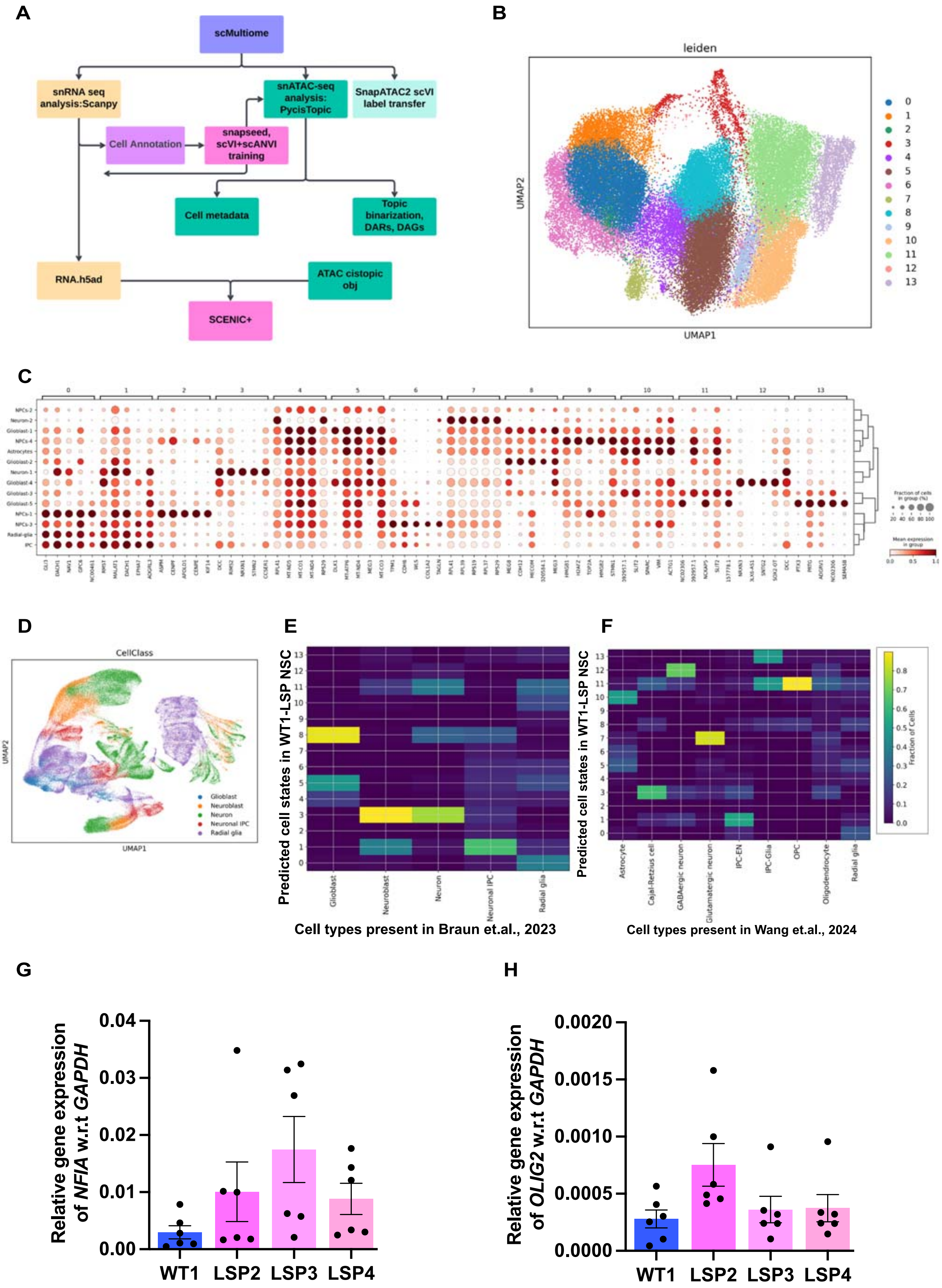
A
scMultiome
snRNA seq analysis:Scanpy
snATAC-seq analysis: PycisTopic
SnapATAC2 scVI label transfer
Cell Annotation
snapseed, scVI+scANVI training
Cell metadata
Topic binarization, DARs, DAGs
RNA.h5ad
ATAC cistopic obj
SCENIC+
B
leiden
UMAP2
UMAP1
0
1
2
3
4
5
6
7
8
9
10
11
12
13
C
D
CellClass
UMAP2
UMAP1
Glioblast
Neuroblast
Neuron
Neuronal IPC
Radial glia
E
Predicted cell states in WT1-LSP NSC
Glioblast
Neuroblast
Neuron
Neuronal IPC
Radial glia
Cell types present in Braun et.al., 2023
F
Predicted cell states in WT1-LSP NSC
Astrocyte
Cajal-Retzius cell
GABAergic neuron
Glutamatergic neuron
IPC-EN
IPC-Glia
OPC
Oligodendrocyte
Radial glia
Fraction of Cells
Cell types present in Wang et.al., 2024
G
Relative gene expression of NFIA w.r.t GAPDH
0.04
0.03
0.02
0.01
0.00
WT1
LSP2
LSP3
LSP4
H
Relative gene expression of OLIG2 w.r.t GAPDH
0.0020
0.0015
0.0010
0.0005
0.0000
WT1
LSP2
LSP3
LSP4

 © The Author(s)

Figure EV3. Overview of single nuclei multiome data analysis.

(**A**) Diagram illustrating the workflow and steps for generating and analyzing single nuclei multiome data. (**B**) UMAP projection showing cell clustering based on the Leiden algorithm using the scanpy pipeline. Each color represents a distinct cell cluster. (**C**) Dot plot displaying expression levels of marker genes across cell clusters generated using scanpy. Dot size indicates the percentage of cells expressing each gene, while color intensity reflects expression level. Upper X-axis shows cell clusters numbered 0–13; Lower X-axis included names of transcripts whose enrichment is being plotted. Y-axis show annotated cell clusters. (**D**) UMAP colored by cell class, highlighting distribution of major cell types such as glioblasts, neuroblasts, neurons, neuronal IPCs, and radial glia; dataset from Braun et al (2023). (**E**) Confusion matrix from scVI-scANVI mapping; Y-axis depicts the 14 unannotated clusters obtained from our multiome analysis; X-axis are the cell types seen in the Braun et al dataset showing prediction accuracy for different cell types across conditions. (**F**) Confusion matrix from scVI-scANVI mapping, detailing prediction outcomes for various cell clusters in Wang et al (2024), dataset. (**G**, **H**) RT-PCR analysis of glia specific transcripts in WT1, LSP2, LSP3 and LSP4 NSC. Each point represents analysis from NSCs generated from multiple neural inductions ($n = 6$) from iPSCs. Y-Axis represents transcript levels for a glial marker mRNA normalized to *GAPDH* levels. (**G**) *NF1A* and (**H**) *OLIG2* transcripts. Error bars: Mean $+/-$ SEM.

A B

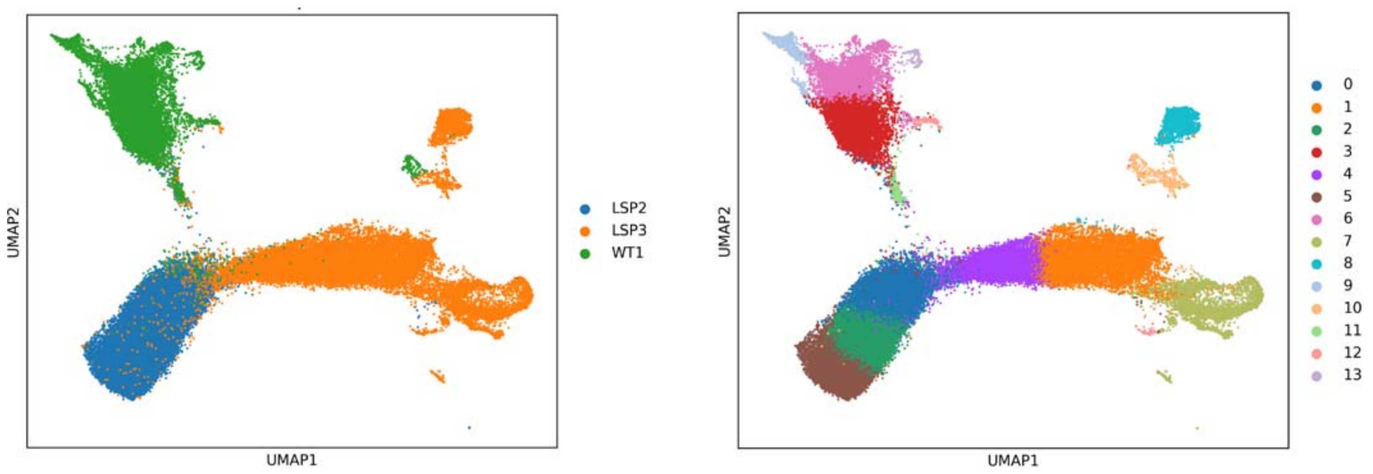

C

Motif logo	TF	Cell-cluster	NES	AUC
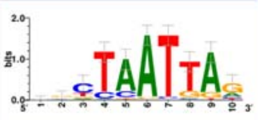	LHX9	Radial-glia	7.269415	0.015211
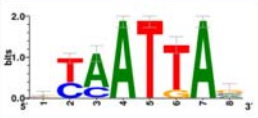	EMX1	Radial-glia	6.380436	0.013670
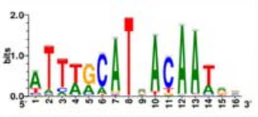	SOX2, POU2F1	Glioblast	7.025670	0.013287
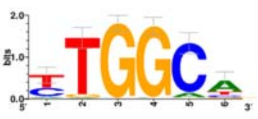	NFIX, NFIC, NFIB, NFIA	Astrocytes	6.759462	0.012162
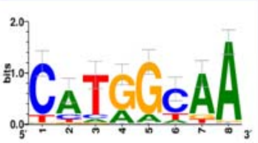	RFX2, RFX3, RFX4, RFX5, RFX1	Astrocytes	18.842255	0.028898

 © The Author(s)

Figure EV4. Validation of gliogenic bias in LSP NSC and transcription factor motif analysis from multiome data.

(**A**) UMAP projection for ATAC-seq dataset showing sample distribution (WT1, LSP2, LSP3) using the snapATAC2 pipeline. (**B**) UMAP projection for ATAC-seq dataset with Leiden clustering across all samples, indicating distinct clusters identified through the snapATAC2 analysis. (**C**) The table describes enriched motifs for each cell cluster obtained through SCENIC+ analysis. Motif logo shows y-axis representing the information content measured in bits, ranging from 0 to 2 bits for DNA sequences. Specifically, a value of 0 bits indicates a position where all nucleotides occur with equal probability. A value of 2 bits indicates a position where only a single nucleotide occurs. The height of each stack shows how conserved that position is - taller stacks mean higher conservation. The x-axis shows the nucleotide positions in the DNA sequence alignment. Each position contains a stack of letters (A, C, G, T) where: the letters are stacked according to their relative frequency at that position. The height of each individual letter within a stack represents how frequently that nucleotide appears at that position and the most common base appears as the largest letter at the top of each stack. In summary, In the motif logo, the x-axis represents sequential nucleotide positions (5′ to 3′), and the y-axis shows information content in bits (0-2.0). Letter height indicates the relative frequency of each nucleotide (A, T, C, G) at each position, with taller letters representing more frequently occurring bases.

© The Author(s)

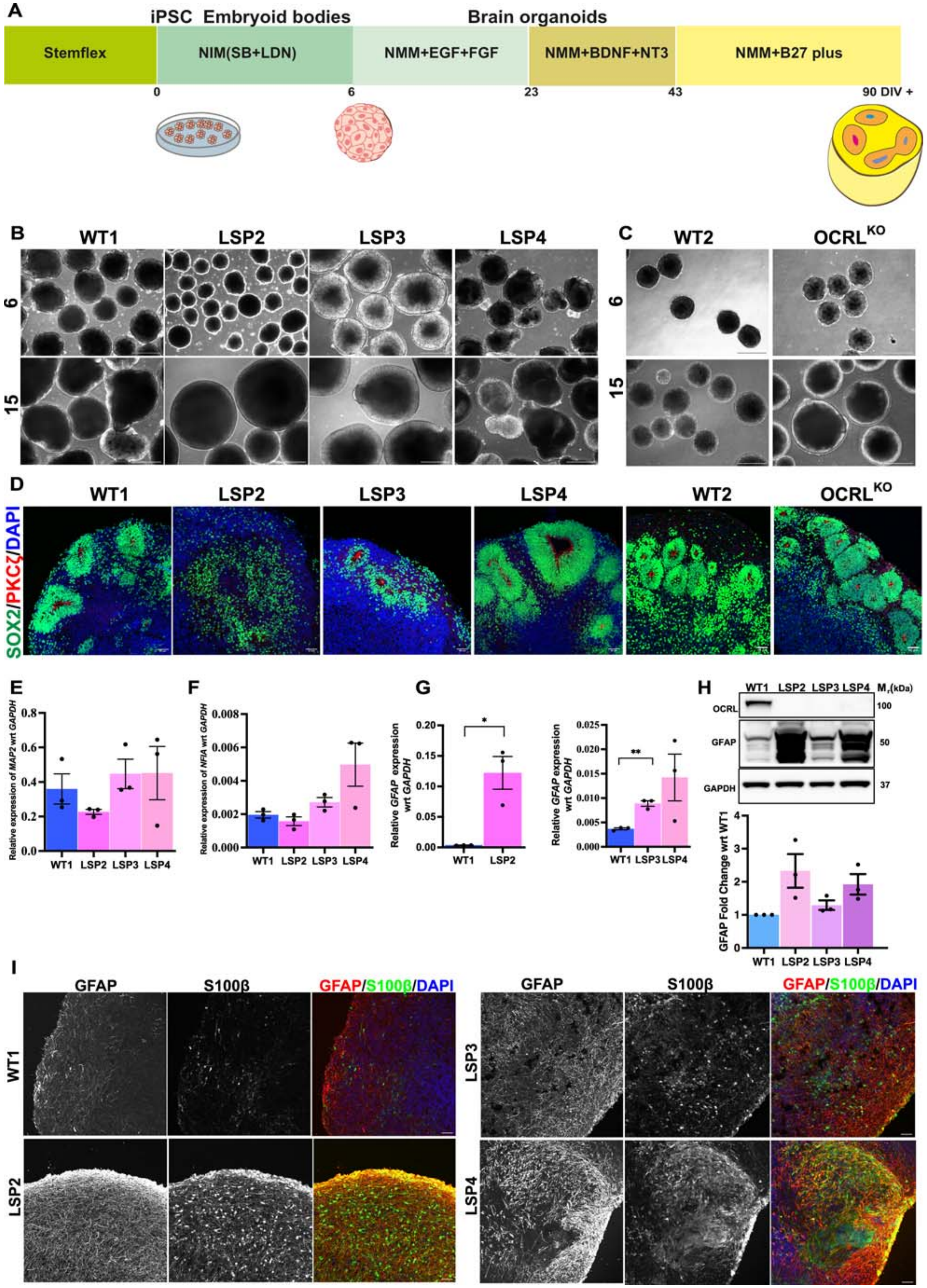
A
iPSC Embryoid bodies
Brain organoids
Stemflex
NIM(SB+LDN)
NMM+EGF+FGF
NMM+BDNF+NT3
NMM+B27 plus
0
6
23
43
90 DIV +
B
WT1
LSP2
LSP3
LSP4
6
15
C
WT2
OCRL^{KO}
D
SOX2/PKCζ/DAPI
E
Relative expression of MAP2 wrt GAPDH
WT1 LSP2 LSP3 LSP4
F
Relative expression of NFIA wrt GAPDH
G
Relative GFAP expression wrt GAPDH
WT1 LSP2
WT1 LSP3 LSP4
H
WT1 LSP2 LSP3 LSP4 M_r(kDa)
OCRL 100
GFAP 50
GAPDH 37
GFAP Fold Change wrt WT1
I
GFAP
S100β
GFAP/S100β/DAPI

Figure EV5. Loss of OCRL results in increased gliogenesis in 90DIV LSP brain organoids.

(**A**) Schematic for brain organoid generation. iPSC are dissociated into single cells to allow formation of embryoid bodies (EBs). EBs are suspended in neural induction medium (NIM) with TGFβ and BMP inhibitors, followed by changing to neural maintenance medium (NMM) with EGF and FGF. For maturation period, the brain organoids are maintained in NMM with BDNF and NT3. (**B**) Phase contrast images showing the morphology of WT1, LSP2, LSP3, LSP4, and (**C**) WT2 and $OCRL^{KO}$ neural spheroids across specific developmental timepoints: 6 and 15 DIV. Scale bar: 500 µm. (**D**) Confocal images of WT1, LSP2, LSP3, LSP4, WT2 and $OCRL^{KO}$ 60DIV showing typical neural rosettes consisting of apical marker protein kinase-C (PKC-ζ, red) and SOX2+ (green) neural stem cells. Nuclei were counterstained with DAPI. Scale bar = 50 µm. RT-PCR analysis of neural spheroids derived from the LSP iPSC lines compared to WT1. Transcript levels for the neuronal marker (**E**) *MAP2* and (**F**) *NFIA* are shown. Y-axis depicts transcript levels normalized to the housekeeping gene GAPDH. Error bars: Mean +/− SEM. (**G**) *GFAP* levels for WT1, LSP2, LSP3, and LSP4 are shown. For *MAP2*, *NFIA* and *GFAP* each point depicts transcripts measured from extracts of 15–20 spheroids ($n = 3$). Error bars: Mean +/− SEM. Statistical test: Unpaired t-test with Welch correction, for *GFAP*, WT1 vs LSP2 $p = 0.0047$*; WT1 vs LSP3 $p = 0.0064$**. (**H**) Western blot of 90DIV neural spheroids cultures WT1, LSP2, LSP3, LSP4; immunoblotting for OCRL (100 kDa) and GFAP (50 kDa) proteins are shown; GAPDH (37 kDa) was used as a loading control. GFAP fold-change in LSP derived 90 DIV neural spheroids analyzed and plotted w.r.t. the control WT1. Y-axis shows the fold change in GFAP levels in LSP relative to WT1 ($n = 3$). Error bars: Mean +/− SEM. (**I**) Individual maximum z-projection of confocal images of GFAP (red) S100β (green) from 90 DIV WT1, LSP2, LSP3 and LSP4 brain organoids. Nuclei were stained with DAPI (blue). Scale bar: 50 µm.

© The Author(s)
